# Levels of Heavy Metals in Popular Cigarette Brands and Exposure to These Metals via Smoking

**DOI:** 10.1100/2012/729430

**Published:** 2012-03-12

**Authors:** Muhammad Waqar Ashraf

**Affiliations:** Department of Mathematics & Natural Sciences, Prince Mohammad Bin Fahd University, P.O. Box 1664, Al Khobar 31952, Saudi Arabia

## Abstract

The levels of selected heavy metals in popular cigarette brands sold and/or produced in Saudi Arabia were determined by graphite furnace-atomic absorption spectrometry (GFAAS). Average concentrations of Cadmium and Lead in different cigarette brands were 1.81 and 2.46 **μ**g g^−1^ (dry weight), respectively. The results obtained in this study estimate the average quantity of Cd inhaled from smoking one packet of 20 cigarettes to be in the range of 0.22–0.78 **μ**g. Results suggest that the quantity of Pb inhaled of smoking one packet of 20 cigarettes is estimated to be 0.97–2.64 **μ**g. The concentrations of Cd and Pb in cigarettes were significantly different between cigarette brands tested. The results of the present study were compared with those of other regional and international studies.

## 1. Introduction

The consumption of tobacco products and number of smokers have been increasing steadily all over the world. The use of cigarettes constitutes one of the major causes of morbidity and mortality in the world. In the tobacco plantation herbicides, insecticides, and fungicides are used to control the various parasites and plant diseases. Tobacco smoke has toxic, genotoxic, and carcinogenic properties. The cigarette smoke contains both organic and inorganic human carcinogenic compounds. Containing 4000 identified chemical compounds, cigarette smoke is very harmful and toxic for human health [1]. Of these toxic materials are heavy metals, particularly cadmium and lead inhaled through.

Several heavy metals found in tobacco smoke such as Cd, Cr, Pb, and Ni also accumulate in tissues and fluids through smoking [2–7]. Tobacco smoking is the most important single source of Cd exposure in the general population. According to Al-Bader et al. [4], the most important sources of Cd in humans are smoking and food. Cadmium can enter the body through tobacco smoking, diet, drinking water, and inhaling it from the air. Small amounts of Cd taken over many years may cause kidney damage and fragile bones, since Cd is mainly stored in bone, liver, and kidneys [8, 9]. Furthermore, Cd causes stomach irritation, vomiting, and diarrhea. Cadmium and lead, present in tobacco smoke, contribute substantially to cancer risk [10]. Cadmium is a group I carcinogen and lead has recently been elevated from a group IIB to a group IIA carcinogen [11].

Cigarette smoke contains substantial amounts of Cd. Average Cd levels in cigarettes range from 1000 to 3000 *μ*g/kg [12–15]. One pack of cigarettes deposits 2–4 *μ*g into the lungs of a smoker while some of the smoke passes into the air to be inhaled by smokers and nonsmokers alike [15, 16], which means, for 20 cigarettes smoked, approximately 2–4 *μ*g of Cd is inhaled by the smoker and as much as a microgram of Cd spreads into the environment. Mussalo-Rauhamaa et al. [17] reported that the mean contents in filter cigarette tobacco sampled from Finland were 1.7 and 2.4 *μ*g^−1^ (dry weight) for Cd and Pb, respectively.

Lead is a highly toxic metal and is capable of causing serious effects on the brain, nervous system, and red blood cells [18–21]. An increase of Pb level is associated with a decrease in the intelligence quotient (IQ) levels and potential behavioral problems [22]. A survey of middle-aged men in 24 British towns showed a strong association between blood Pb concentrations and alcohol and cigarette smoking [23]. Smoking of 20 cigarettes a day has been estimated to result in the inhalation of 1–5 *μ*g Pb [20]. The WHO estimates 2–6% of Pb in cigarettes is inhaled by the smoker [21]. It was reported that Pb in tobacco has been associated with impaired fetal growth and brain development [24].

The consumption of tobacco products and the number of smokers have been increasing steadily throughout the world and Saudi Arabia is no exception to this. Tobacco in Saudi Arabia continued to display high growth in 2010, rising significantly in both volume and value terms. Unlike Western markets, in which smoking rates are on the decline as a result of the many concerted initiatives aimed at reducing smoking, Saudi Arabia's smoking population is rising. Pipe tobacco remains the dominant subcategory, which is unsurprising in light of the shisha culture that is deeply embedded across the Middle East (http://www.euromonitor.com/Tobacco_in_Saudi_Arabia). In an earlier report it was revealed that the overall prevalence of smoking was 21.1% for males and 0.9% for females. Most smokers (78%) were young to middle-aged (21–50 years old). Smoking prevalence was higher among married people, uneducated people, and those in certain occupations: manual workers, businessmen, army officers, and office workers [25].

The objectives of the present study were manyfold: first, to investigate Cd and Pb concentrations in different brands of tobacco cigarettes sold and/or produced in Saudi Arabia; second, to find out if there are significant differences between different cigarette brands in their heavy metal contents; third, to estimate their (Cd and Pb) amounts in the mainstream smoke according to Mussalo-Rauhamaa et al. [17] study; fourth, to compare our data with the one published for other parts of the world.

## 2. Materials and Methods

Graphite furnace atomic absorption spectrometry (GFAAS) (Shimadzu AA-6200 equipped with ASC 6100 autosampler) was used for the determination of Cd and Pb. The wavelengths for Cd and Pb were set to 228.8 and 283.3 nm, respectively and spectral bandpass to 0.7 nm. All reagents used were of Specpure grade in quality. Standard solutions of 1000 ppm for Cd and Pb (E.Merck), 35–38% HCl, 70% HNO_3_ (Specpure, E.Merck) were used. Glassware and PE containers were soaked in 5% nitric acid for 24 h, cleaned with deionized water, and dried in such a manner to ensure that any contamination from glassware does not occur.

Twenty different brands of cigarettes were purchased randomly from local market in Eastern Province, Saudi Arabia. Composites were a homogenized mixture prepared by removing the papers and filters of 20 cigarettes taken randomly from four different batches (5 cigarettes from each pack of different batch number). Care was taken to avoid any source of contamination, and this preparation was carried out in a clean environment. The weight of tobacco mixtures was then measured ranging from 600 to 700 mg per cigarette.

The method used in this study is applicable to the determination of Cd and Pb in cigarettes by GFAAS. Tobacco samples were placed and spread in covered clean glass containers until they became dry. Thus, care was taken that the samples were not directly influenced by dust during air drying. For analysis of Cd and Pb, about 0.5 g of air-dried tobacco sample was placed in a PTFE vessel and allowed to digest with a mixture of HNO_3_ and HCl with a ratio of 8:2 v/v by heating the PTFE vessel in a water bath-shaker for 5 h at 100°C. After cooling, 10 mL of deionized water was added, and the solution was then filtered through a Whatman filter paper 40 into a 25 mL volumetric flask. The volume obtained was topped up to the mark with deionized water [26]. Quantification was achieved by interpolating the relevant calibration curves prepared from aqueous solutions of metal standards in the same acid concentration, in order to minimize matrix effects.

To validate and confirm the reliability of the method used for the analysis of Cd and Pb in cigarettes, two certified standard reference materials NIST-1575a (pine needles) and NIST-1570 (Spinach leaves) were analyzed taking into consideration the reproducibility and accuracy of the results obtained by the acid digestion method. Results obtained for the standard reference materials are displayed in Table 1.

The results were in good agreement with the certified values for Pb and Cd. Moreover, the precision, accuracy, and reproducibility of results for every run was started with a control blank and testing several quality control (QC) solutions. This procedure was repeated after every seven samples. Results were within 3% of QC values. For every sample five replicates were taken and the average value was calculated. The results were statistically analyzed using ANOVA and Student's *t*-test (Statistica 5.0). Significant differences were found between different cigarette brands in Cd and Pb contents. ANOVA analysis for Cd and Pb shows that there are significant differences in the concentrations of the 20 different cigarette brands. The obtained values for Cd and Pb in the two reference materials are in consistence with their certified values.

## 3. Results and Discussion

The results of Cd and Pb concentrations, together with other relevant details for tobacco materials sold and produced in Saudi Arabia, are given in Tables 2 and 3. The average concentration of Cd in cigarettes tested is 1.81 *μ*g g^−1^ (dry weight) ranging from 0.83 to 2.78 *μ*g g^−1^. This finding is in agreement with Watanabe et al. [14], who reported that Cd content in cigarettes sampled from various countries ranged from 0.29 to 3.38 *μ*g g^−1^. Compared with the reported results for Cd in the UK (0.90 *μ*g g^−1^) and Korean cigarettes (1.02 *μ*g g^−1^), Cd contents in the brands studied are double and similar, respectively [27].

Lead concentrations in cigarette brands studied, ranged from 1.33 to 3.61 *μ*g g^−1^ dry weight with an average of 2.46 *μ*g g^−1^. These results obtained for Pb are in agreement with those results reported by Watanabe et al. [14] that Pb content in cigarettes sampled from various countries ranged from 0.46 to 3.66 *μ*g g^−1^. Compared with the Pb contents reported in the UK (1.35 *μ*g g^−1^) and Korea (0.74 *μ*g g^−1^), the average Pb contents in cigarette brands studied are 1.5 and 3.5 times higher, respectively. Our results are also comparable with the data reported for cigarettes produced and consumed in Jordon, Cd at the level of 2.64 *μ*g g^−1^ and Pb at 2.67 *μ*g g^−1^ [28]. According to a Reuters report, a recent tobacco study conducted by researchers from the Buffalo-based Roswell Park Cancer Institute found that cigarettes produced in China contain three times the amount of heavy metals found in Canadian manufactured brands (http://www.whatsonxiamen.com/news15008.html).

 It has been documented in the literature that an average of 2.0 and 5.8% of Cd and Pb, respectively, contained in cigarettes is passed to mainstream smoke [17]. Using this fact in the present study, the amounts of Cd contained in 20 cigarettes passed to mainstream smoke ranged from 0.22 to 0.78 *μ*g with an average of 0.48 *μ*g. The details are presented in Table 2.  Table 3 furnished information about average amounts of Pb contained in 20 cigarettes which passed to mainstream smoke. On average the estimated amount of Pb in stream smoke was to be 2.4 (0.97–1.87) *μ*g.

It is generally accepted that Cd and Pb concentrations in cigarettes range from 1 to 3 and 1 to 2 *μ*g g^−1^, respectively [14, 17]. It was reported that Cd and Pb concentrations in filter cigarettes were 1.7 and 2.4 *μ*g g^−1^, respectively [17]. Tobacco smoking is the most significant single source of Cd exposure in the general population. On average, cigarettes contain 1-2 *μ*g Cd. It can be estimated that a person smoking 20 cigarettes per day takes about up to 1 *μ*g of Cd per day. For comparison it can be mentioned that the concentration of Cd in ambient air generally is below 5 ng/m^3^, and in most cases less than 0.01 *μ*g Cd in airborne origin is absorbed in the lungs daily [13]. Tobacco grown in soils with higher available cadmium and lead levels has correspondingly higher levels in tobacco lamina. Thus, cigarette brands with similar tar deliveries could yield markedly different smoke particulate levels of heavy metals depending on where the tobacco was grown and filter ventilation [29].

There is no sufficient data about the heavy metal concentrations in cigarette brands in Saudi Arabia including Cd and Pb. This study provides a new data for the health authorities such as the Ministry of Health, Ministry of Environment and other world health authorities such as the UNICEF and WHO. Moreover, the results obtained give very important information for the smokers in Saudia to know that Cd and Pb are toxic pollutants affecting adversely on their health besides the other toxic chemicals present in cigarettes such as nicotine.

This study confirms that tobacco is a notable source of many heavy metal pollutants particularly Cd and Pb. The amount of Cd inhaled from smoking one pack of 20 cigarettes of different cigarette brands is estimated to be 1.40–2.70 *μ*g. This value is comparable with the values from UK cigarettes (1.32–2.64 *μ*g) and Korean cigarettes (1.54–3.08 *μ*g). The small variation could be possibly attributed to Cd soil content, type of tobacco, growth conditions, and tobacco treatment process. The amount of Pb inhaled from smoking one pack of 20 cigarettes of the brands studied is estimated to be 1.98–3.37 *μ*g, and this value is nearly 4 times higher compared with the UK cigarettes (0.22–0.65 *μ*g) and 3.5 times that of Korean cigarettes (0.4–1.19 *μ*g). Smoking of 20 cigarettes per day has been estimated to result in the inhalation of 2–4 *μ*g Cd and 1–5 *μ*g Pb, or even more [16, 28, 30].

## Figures and Tables

**Table 1:** Results obtained for the standard reference materials together with certified value.

SRM	Element	Certified value (*μ*g g^−1^)	Measured value
Pine needles	Pb	0.167 ± 0.013	0.161 ± 0.102
NIST-SRM 1575a	Cd	0.233 ± 0.009	0.214 ± 0.013
Spinach leaves	Pb	0.200 ± 0.006	0.198 ± 0.012
NIST-SRM 1570	Cd	2.890 ± 0.070	2.830 ± 0.095

**Table 2:** The weight of 20 cigarettes (g) concentration of Cd in *μ*g g^−1^ (dry weight) and average estimated amount of Pb passed to main stream smoke for 20 cigarettes a day in 20 different cigarette brands.^a^

No.	Brand name	Weight of 20 cigarettes (g)	Mean ± S.D. (*μ*g g^−1^)	Estimated amount of Cd in stream smoke (*μ*g)
(1)	Gold Coast	12.82	1.97 ± 0.04	0.51
(2)	Monte Carlo	13.95	2.66 ± 0.09	0.74
(3)	Gauloises	13.85	1.30 ± 0.03	0.36
(4)	Winston	12.75	2.60 ± 0.07	0.66
(5)	Dunhill	11.95	0.97 ± 0.05	0.23
(6)	Salem	13.72	1.93 ± 0.05	0.53
(7)	Merit	12.70	1.53 ± 0.03	0.39
(8)	Gitanes	12.87	2.51 ± 0.02	0.65
(9)	Camel	12.30	0.91 ± 0.07	0.22
(10)	Marlboro	11.77	0.78 ± 0.02	0.18
(11)	Kent	14.12	0.83 ± 0.06	0.23
(12)	Wills	12.35	1.73 ± 0.04	0.43
(13)	Parliament	12.61	2.13 ± 0.02	0.54
(14)	Carlton	14.66	2.58 ± 0.05	0.76
(15)	Garam	12.55	1.95 ± 0.03	0.49
(16)	Gold Leaf	12.89	2.11 ± 0.07	0.54
(17)	Davidoff	13.17	0.92 ± 0.07	0.24
(18)	Vogue	12.83	2.40 ± 0.04	0.62
(19)	Rothman	13.99	2.78 ± 0.07	0.78
(20)	L & M	13.10	2.13 ± 0.03	0.56

^a^The results were calculated for five replicate determinations.

**Table 3:** The weight of 20 cigarettes (g) concentration of Pb in *μ*g g^−1^ (dry weight) and average estimated amount of Pb passed to main stream smoke for 20 cigarettes a day in 20 different cigarette brands.^a^

No.	Brand name	Weight of 20 cigarettes (g)	Mean ± S.D. (*μ*g g^−1^)	Estimated amount of Pb in stream smoke (*μ*g)
(1)	Gold Coast	12.82	2.83 ± 0.08	2.10
(2)	Monte Carlo	13.95	2.17 ± 0.04	1.75
(3)	Gauloises	13.85	2.33 ± 0.05	1.87
(4)	Winston	12.75	1.96 ± 0.06	1.45
(5)	Dunhill	11.95	1.88 ± 0.08	1.30
(6)	Salem	13.72	2.06 ± 0.05	1.64
(7)	Merit	12.70	2.25 ± 0.03	1.65
(8)	Gitanes	12.87	2.10 ± 0.08	1.57
(9)	Camel	12.30	1.99 ± 0.07	1.42
(10)	Marlboro	11.77	1.55 ± 0.05	1.06
(11)	Kent	14.12	2.60 ± 0.06	2.13
(12)	Wills	12.35	2.15 ± 0.04	1.54
(13)	Parliament	12.61	3.61 ± 0.03	2.64
(14)	Carlton	14.66	2.95 ± 0.04	2.50
(15)	Garam	12.55	1.33 ± 0.03	0.97
(16)	Gold Leaf	12.89	2.97 ± 0.06	2.22
(17)	Davidoff	13.17	3.14 ± 0.07	2.34
(18)	Vogue	12.83	3.55 ± 0.08	2.64
(19)	Rothman	13.92	2.58 ± 0.05	2.08
(20)	L & M	13.10	3.28 ± 0.09	2.49

^a^The results were calculated for five replicate determinations.
